# Associations Between Comorbidities, Developmental Status, and Disease Severity in Children With Autism Spectrum Disorder: A Multicenter Cross‐Sectional Study in China

**DOI:** 10.1002/aur.70253

**Published:** 2026-04-13

**Authors:** Dizhou Pang, Guiqin Duan, Qing Shang, Huichun Zhang, Binbin Wang, Huaili Ding, Huihui Kang, Yue Zhao, Yuxia Lu, Gai Tian, Li Zhang, Wu Jiang, Ganyu Wang, Cailing Liang, Lingling Zhang, Bingbing Li, Zhenghua Li, Chunlan Song, Yanyan Yang, Yazhe Wang, Kaichen Yue, Yongbin Lang, Jiatian Liu, Xiaoli Zhang, Yiran Xu, Changlian Zhu

**Affiliations:** ^1^ Department of Child Development and Behavior The Third Affiliated Hospital of Zhengzhou University Zhengzhou China; ^2^ Henan Key Laboratory of Child Brain Injury and Henan Pediatric Clinical Research Center Institute of Neuroscience and the Third Affiliated Hospital of Zhengzhou University Zhengzhou China; ^3^ Rehabilitation Center, Henan Children's Hospital Zhengzhou China; ^4^ Child Health Department Zhoukou Maternal and Child Healthcare Hospital Zhoukou China; ^5^ Department of Rehabilitation Medicine Fujian Children's Hospital Fuzhou China; ^6^ Child Rehabilitation Department Xinyang Central Hospital Xinyang China; ^7^ Children Rehabilitation Department Puyang Maternity and Child Healthcare Centers Puyang China; ^8^ Child Rehabilitation Department Maternity and Child Healthcare Hospital of Dingzhou Dingzhou China; ^9^ Children Rehabilitation Department Xingyang People's Hospital Zhengzhou China; ^10^ Child Health Department Nanning Maternal and Child Healthcare Hospital Nanning China; ^11^ Center for Brain Repair and Rehabilitation, Institute of Neuroscience and Physiology, University of Gothenburg Gothenburg Sweden

**Keywords:** autism spectrum disorder, childhood autism rating scale, comorbidities, disease severity, Gesell developmental schedule, intellectual developmental disorders, Wechsler intelligence scales

## Abstract

Children with autism spectrum disorder (ASD) frequently present with co‐occurring conditions that can influence autism symptom severity and complicate clinical management. However, studies with clinician‐confirmed diagnoses in non‐Western populations remain limited. In this multicenter cross‐sectional study, 1279 children aged 3–14 years with DSM‐5‐confirmed ASD were recruited from eight medical centers in China. Autism symptom severity was assessed using the Childhood Autism Rating Scale (CARS). Comorbidities were identified through clinical evaluation and specialized assessments, and developmental status was measured using the Gesell Developmental Schedule (GDS) and Wechsler Intelligence Scales. Associations with CARS scores were analyzed using generalized linear regression. Of participants, 96.6% had at least one comorbidity and 71.2% had multiple comorbidities. Common conditions were intellectual developmental disorders (IDD) (87.3%), food selectivity (45.3%), insomnia disorder (16.9%), developmental regression (15.6%), and behavioral problems (14.6%). Patterns differed by sex and age: gastrointestinal problem and sleep‐related interventions were more common in girls, whereas food selectivity was more common in boys. Older children showed higher rates of tic disorders, asthma, epilepsy, and offensive language, although these findings should be interpreted cautiously because the subgroup aged ≥ 6 years was small. In adjusted analyses, IDD, food selectivity, pica, insomnia disorder, and developmental regression were associated with higher CARS scores, whereas higher GDS and Wechsler scores were associated with lower CARS scores. In this Chinese cohort, comorbidities were prevalent and showed distinct sex‐ and age‐related patterns. Several comorbidities were associated with greater autism symptom severity, underscoring the importance of comprehensive developmental and medical assessment in ASD care.

## Lay Summary

This large study found that nearly all children with ASD in this large clinical sample had at least one additional condition, such as intellectual disability (ID), sleep problems, or selective eating. These co‐occurring conditions were associated with more severe autism symptoms, while better cognitive understanding linked to milder symptoms. The findings highlight the clinical importance of comprehensive developmental and medical evaluation in ASD care.

## 1 Introduction

Autism spectrum disorder (ASD) is a group of heterogeneous neurodevelopmental conditions characterized by difficulties in social communication, restricted interests, and repetitive behaviors (APA. 2013). ASD affects individuals worldwide, and its prevalence has been increasing over the past few decades (Maenner et al. 2023). Alongside the core symptoms of ASD, children frequently present with a range of comorbid conditions, complicating both diagnosis and clinical management (Hyman et al. 2020). These comorbidities include intellectual developmental disorders (IDD), gastrointestinal problems, insomnia, epilepsy, and a variety of psychiatric or behavioral symptoms (Micai et al. 2023; Neumeyer et al. 2019).

Population‐based studies indicate that the majority of individuals with ASD experience multiple co‐occurring conditions, often spanning psychiatric and medical domains (Lundström et al. 2015; Simonoff et al. 2008). The presence of such co‐occurring conditions can significantly reduce quality of life and increase caregiver burden. Timely and accurate identification of comorbidities is therefore critical to improving outcomes and tailoring interventions in ASD care (Hyman et al. 2020; Matson and Nebel‐Schwalm 2007).

Despite growing recognition of the comorbidity burden, several limitations characterize the existing literature. Many large‐scale studies rely on parental reports, administrative databases, or retrospective records, which may lack standardized multidisciplinary clinical confirmation. In addition, most evidence derives from Western populations, limiting generalizability to non‐Western contexts. This geographic concentration leaves important gaps in understanding comorbidity patterns in Asian populations, where healthcare systems, referral pathways, and early screening practices may differ.

In China, relatively few large‐scale studies have examined comorbidities in children with ASD using direct clinician‐confirmed diagnoses based on standardized protocols. Furthermore, there is limited information on how these comorbidities differ by age or sex in Asian populations. Another understudied area is the relationship between comorbidities and ASD severity. While several studies suggest that conditions like intellectual disability (ID) or behavioral problems may worsen ASD symptoms (Matson and Shoemaker 2009), few have quantified these associations using validated severity scales like the Childhood Autism Rating Scale (CARS) in large, diverse clinical samples.

To address these gaps, the current multicenter cross‐sectional study aims to: (1) assess the prevalence and types of comorbidities in children with ASD across eight medical centers in China; (2) examine age‐ and sex‐specific differences in comorbidity profiles; and (3) evaluate how comorbidities and developmental levels (assessed by the Gesell Developmental Schedule (GDS) and Wechsler Intelligence Scales) relate to ASD symptom severity, as measured by CARS. By providing high‐quality, clinician‐confirmed data from a large and geographically diverse pediatric cohort, this study seeks to inform early detection strategies, promote individualized interventions, and support the development of culturally tailored clinical guidelines for ASD in China.

## 2 Methods

### 2.1 Study Design and Setting

This was a multicenter cross‐sectional study conducted from March 2022 to December 2023. The primary objective was to investigate the prevalence and patterns of comorbidities and their associations with autism severity in pediatric patients diagnosed with ASD across multiple regions in China.

Medical institutions were screened for eligibility based on predefined inclusion criteria: (1) clinical qualification to diagnose ASD based on DSM‐5; (2) capacity to perform or coordinate multidisciplinary evaluation of comorbidities; (3) experience with standardized intervention techniques; and (4) inclusion of different levels of hospitals, including provincial, city, and town hospitals. Ultimately, eight centers were selected across three provinces, representing a mix of urban and semi‐urban regions. All were affiliated with general hospitals, not standalone clinics, and offered outpatient and inpatient pediatric services.

### 2.2 Participants

Children aged 3–14 years with a clinical diagnosis of ASD were eligible for inclusion. ASD was diagnosed by experienced developmental‐behavioral pediatricians or psychiatrists according to the DSM‐5 criteria. Exclusion criteria included: Known syndromic or genetic disorders (e.g., Fragile X syndrome, Rett syndrome); Severe sensory impairments (e.g., severe blindness or deafness); Uncontrolled epilepsy or acute febrile seizures; Major physical disabilities interfering with assessment; Ongoing acute psychiatric crisis or declined consent.

**FIGURE 1:**
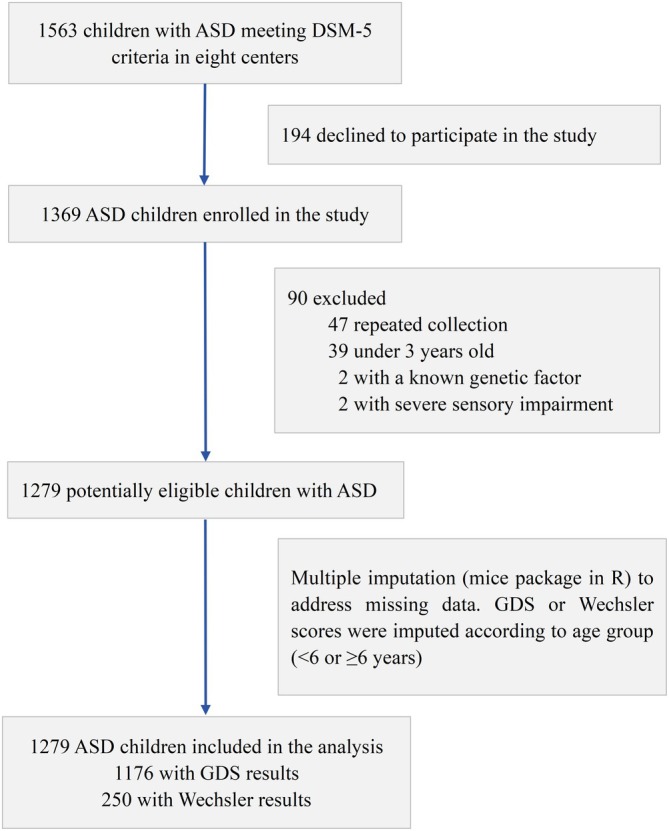
Flow diagram of included and excluded participants in the study. The diagnosis of ASD was determined using the DSM‐5 diagnostic criteria and scores from various assessment tools, including the Childhood Autism Rating Scale (CARS), Autism Diagnostic Observation Schedule (ADOS), Autism Behavior Checklist (ABC), and other scales. Wechsler results included: WPPSI (Wechsler Preschool and Primary Scale of Intelligence), WISC‑III (Wechsler Intelligence Scale for Children III), and WISC‑IV (Wechsler Intelligence Scale for Children IV). Among 1279 children with ASD, 147 had both GDS and Wechsler intelligence test results available. ASD, autism spectrum disorder; GDS, Gesell Developmental Schedule.

Patients were recruited through consecutive sampling during routine diagnostic visits at each center. Families were approached by trained clinical staff and provided with detailed information about the study. Of the 1563 children contacted, written informed consent was obtained for 1369. Ultimately, 1279 children were included in the analysis. Reasons for exclusion after obtaining informed consent were as follows: repeated attendance at different centers within the same region (*n* = 47), age under 3 years (*n* = 39), genetic disorders (*n* = 2), and visual or hearing impairments (*n* = 2). The complete participant flow is detailed in Figure 1.

### 2.3 Standardization and Quality Control Across Centers

To ensure diagnostic consistency and data reliability across all participating centers, inter‐rater calibration sessions were conducted prior to study initiation to enhance scoring consistency. We maintained rigorous, multi‐faceted quality control procedures throughout the study.

#### 2.3.1 Protocol Development and Pre‐Study Training

Prior to participant enrollment, all investigators and assessors from the eight centers attended a centralized training workshop. A detailed common study manual, covering all protocols and forms, was distributed to each site and served as the primary study reference. Subsequently, a unified training session was organized and led by the coordinating center (The Third Affiliated Hospital of Zhengzhou University) for all involved personnel. To further ensure consistency, inter‐rater reliability exercises and cross‐validation checks were implemented across centers, thereby securing uniformity and stability in data collection procedures.

#### 2.3.2 Data Collection and Ongoing Quality Control

The diagnosis of ASD was confirmed at each center by experienced developmental‐behavioral pediatricians or child psychiatrists in accordance with DSM‐5 criteria. The assessment of comorbidities was supported by a core multidisciplinary team, which included pediatric specialists in respiratory, gastroenterology, neurology, and other relevant fields as needed. To ensure diagnostic consistency and resolve uncertainties, the coordinating center conducted regular online case conferences where complex cases were reviewed and discussed collectively. All submitted materials underwent initial quality review, and any issues identified were promptly returned to the respective centers for correction or supplementation.

#### 2.3.3 Final Quality Control and Comprehensive Review

After data collection, a final quality control audit was performed to verify and rectify any unresolved issues from the initial review. Subsequently, a comprehensive, center‐specific evaluation of key metrics (data volume, completeness, comorbidity records) followed and disseminated the detailed results to all participating centers to ensure transparency.

### 2.4 Assessment of Comorbidities

Comorbidities were assessed through direct clinical examination, caregiver interviews, and appropriate testing based on standardized diagnostic guidelines. All diagnoses were made through the collaboration of a multidisciplinary team at each center. This team was led by the department of child developmental and behavioral and comprised specialists from pediatric psychiatry, pulmonology, gastroenterology, neurology, rheumatology/immunology, and among other relevant subspecialties.

#### 2.4.1 Mental Disorders

Intellectual developmental disorders (IDD), including ID and global developmental delay (GDD), were diagnosed according to DSM‐5 criteria. ID was defined as an intelligence quotient (IQ) < 70 assessed using the Wechsler Intelligence Scales. GDD was identified as a developmental quotient (DQ) ≤ 75 in two or more domains of the GDS (APA. 2013; Burack et al. 2021). Insomnia disorder was diagnosed based on sleep status evaluated via caregiver questionnaire and clinical interview, following ICD‐10 criteria (Riemann et al. 2022). Pica and tic disorders were diagnosed per DSM‐5, supported as needed by cranial MRI, serum ceruloplasmin, and trace element tests (APA. 2013; Schnitzler 2022; Singer 2019).

#### 2.4.2 Physical Comorbidities

Overweight and obesity were classified using WHO BMI‐for‐age z‐score cutoffs: overweight (≥ +1 to < +2) and obesity (≥ +2) (Buoncristiano et al. 2021). Gastrointestinal symptoms (e.g., constipation, bloating, diarrhea) were assessed through clinical examination, symptom history, and relevant investigations such as stool tests or abdominal ultrasound, consistent with established guidelines (Drossman 2016; Hyams et al. 2016). Allergic diseases (e.g., eczema, asthma, allergic rhinitis) were diagnosed using allergen testing, lung function evaluation, and immune examinations (Bieber 2008; Bousquet et al. 2020; Danvers et al. 2020; Gaillard and Moeller 2021; Pillebout and Sunderkötter 2021). Febrile seizures and epilepsy were diagnosed based on standard neurological guidelines, incorporating EEG, cranial MRI, and clinical history (Guerrini 2006; Subcommittee on Febrile Seizures 2011).

#### 2.4.3 Other Problems

We also investigated clinically significant symptoms that, while not meeting formal diagnostic criteria for specific disorders, are prevalent and impactful in ASD. These include food selectivity (defined as persistent refusal of specific food categories for > 3 months), behavioral problems (including but not limited to aggression [e.g., hitting, kicking, biting others], self‐injurious behavior [e.g., head‐banging, hand‐biting, excessive scratching], property destruction, and severe tantrums), developmental regression (defined as a loss of established skills for > 3 months), swallowing/chewing problems, and offensive language. A child was coded as positive for behavioral problems when one or more challenging behaviors were repeatedly present, as confirmed by caregiver interview and clinical observation, and were judged by the clinician to be of sufficient frequency and severity to cause functional impairment, safety concerns, or the need for specific management/intervention. Such symptoms can cause significant caregiver distress and may represent precursors to comorbid conditions (Gaitanis et al. 2023; Kanne and Mazurek 2011; Leader et al. 2020; Malhi et al. 2019; Sharp et al. 2013). Standardized assessment protocol for comorbidities is provided in Table S1.

### 2.5 Developmental Status Measurement

The GDS was administered to children under 6 years of age to assess developmental status across five domains: adaptive behavior, gross motor, fine motor, language, and personal‐social behavior. For children aged 4.5 years and older, as well as adolescents, who were deemed to have sufficient cognitive and comprehension abilities, cognitive function was evaluated using the Wechsler Preschool and Primary Scale of Intelligence (WPPSI), the Wechsler Intelligence Scale for Children‐III (WISC‐III), or the WISC‐IV, as appropriate for their age. Furthermore, some centers employed additional assessment tools tailored to the specific conditions of the patients, such as the 0–6 Year Old Children's Neuropsychological Development Scale, a tool developed for Chinese children. For statistical analyses, only GDS and Wechsler scores were included to ensure cross‐center comparability. Additional tools were used for clinical purposes only.

### 2.6 Autism Severity Measurement

Autism severity was measured using the CARS, which includes 15 items rated on a four‐point scale. In this study, CARS was used only as a measure of symptom severity rather than a diagnostic tool. Participants with CARS scores below 30 were still included if they met diagnostic criteria. Total score ranges from 30 to 36 points, with fewer than five items scoring below three points indicating mild–moderate autism, and a score of > 36 points with a minimum of five items scoring above three points suggesting severe autism (Schopler et al. 1980). Other tools such as the Autism Diagnostic Observation Schedule (ADOS), the Autism Behavior Checklist (ABC), or the Checklist for Autism in Toddlers (CHAT) were used as needed based on clinical judgment.

### 2.7 Covariates

Covariates included in regression models were selected based on previous literature linking them to autism severity. These included: sex, age, gestational hypertension, premature birth, paternal age at conception, and family history of mental illness (Elgen et al. 2023; Rieske and Matson 2020; Wallace et al. 2008). Additional covariates such as food selectivity, developmental regression, and offensive language were included in later models due to their observed impact on symptom presentation.

### 2.8 Statistical Analysis

All statistical analyses were performed using R software (version 4.4.3; R Foundation for Statistical Computing, Vienna, Austria). Statistical significance was defined as a two‐sided *p* < 0.05.

The proportion of missing data ranged from 0.2% to 20.4% across study variables. Missingness primarily arose from age‐dependent assessment eligibility (e.g., GDS for younger children and Wechsler scales for older children) and protocol variability across centers. Missing data were addressed using multiple imputation by chained equations (MICE) implemented with the mice package in R. Twenty imputed datasets were generated, with 50 iterations performed per imputation to ensure convergence. Predictive mean matching was used for continuous variables, and logistic regression models were applied for binary variables. The imputation model included all variables used in subsequent regression analyses, including: CARS scores, comorbidity indicators, developmental measures (GDS domains and Wechsler scores), demographic covariates (age, sex), perinatal and familial covariates.

To maintain developmental consistency, imputation was stratified by age group. For children < 6 years, GDS scores were imputed with available Wechsler data informing the imputation where applicable. For children ≥ 6 years, Wechsler scores were imputed using available GDS results under the same principle. This approach ensured alignment between developmental metrics and IDD classification.

Convergence diagnostics were visually inspected to confirm the stability of imputation chains. Parameter estimates from the imputed datasets were pooled using Rubin's rules to obtain final regression coefficients and standard errors. As sensitivity analyses, we conducted the following: a complete‐case analysis, as well as subgroup analyses restricted to children aged < 6 years and those aged ≥ 6 years. The results of these sensitivity analyses are presented in Tables [Link], [Link].

Continuous variables were summarized as means ± standard deviations (SD) if normally distributed, or medians with interquartile ranges (IQR) if skewed. Group comparisons were performed using independent‐sample *t*‐tests or Mann–Whitney *U* tests as appropriate. Categorical variables were summarized as frequencies and percentages and compared using chi‐square tests, with Fisher's exact test applied when expected frequencies were < 5.

Associations between comorbidities, developmental status, and autism severity (CARS total score) were examined using generalized linear models (GLMs) with Gaussian distribution and identity link function, equivalent to linear regression modeling. This approach was chosen because CARS scores are continuous and approximately normally distributed.

Three sequential models were constructed for the analysis of comorbidities and CARS scores: Model 1: Unadjusted; Model 2: Adjusted for age and sex; Model 3: Further adjusted for premature birth, paternal age at conception, gestational hypertension, and family history of mental illness.

For analyses examining developmental measures and CARS scores: Model 1: Unadjusted; Model 2: Adjusted for age, sex, premature birth, paternal age at conception, gestational hypertension, and family history of mental illness; Model 3: Additionally adjusted for selected comorbidities associated with symptom presentation (food selectivity, developmental regression, offensive language, pica, and insomnia disorder).

Regression coefficients (*β*) with 95% confidence intervals (CI) were reported. To minimize multicollinearity, developmental domain scores were analyzed in separate models. Variance inflation factors (VIFs) were examined and confirmed to be within acceptable limits (< 5).

## 3 Results

### 3.1 Participant Characteristics

This study included 1279 children with ASD (median age: 4.1 years, IQR: 3.4–5.1; 81.3% male), 89.2% of whom were under 6 years old. The median CARS score was 34 (IQR: 32–37), with 49 (3.8%) scoring < 30, 877 (68.6%) scoring 30–36, and 353 (27.6%) scoring >36. GDS assessments (*n* = 1176) showed a mean Adaptive Behavior Developmental DQ of 54.3 (SD 14.4), and 65 (IQR: 55–75), 59 (47–71), 35 (24–47), and 48 (40–56) for Gross Motor, Fine Motor, Language, and Personal‐social subscales, respectively. Among the 250 children assessed with Wechsler Intelligence Scales (Wechsler assessments were conducted primarily in children ≥ 4.5 years with sufficient cognitive capacity), 32 (12.8%) fell within the normal range, 31 (12.4%) were borderline, and 187 (74.8%) were classified as having ID (see Table 1 for further details).

**TABLE 1:** General characteristics of the participants in the study.

		Total	Sex		*p*	Age		*p*
			Male	Female		< 6 years	≥ 6 years	
Basic information (*n* = 1279)	Number of cases, *n* (%)	1279 (100.0%)	1040 (81.3%)	239 (18.7%)	—	1141 (89.2%)	138 (10.8%)	—
	Age, median (IQR)	4.1 (3.4, 5.1)	4.2 (3.4, 5.2)	4.1 (3.5, 4.8)	0.547	4.0 (3.3, 4.8)	6.7 (6.3, 7.0)	—
	Premature birth, *n* (%)	96 (7.5%)	82 (7.9%)	14 (5.9%)	0.284	85 (7.4%)	11 (8.0%)	0.826
	Gestational hypertension, *n* (%)	57 (4.5%)	47 (4.5%)	10 (4.2%)	0.821	55 (4.8%)	2 (1.4%)	0.070
	Family history of mental illness, *n* (%)	26 (2.0%)	21 (2.0%)	5 (2.1%)	1.000	21 (1.8%)	5 (3.6%)	0.190
	Paternal age at conception, median (IQR)	29.0 (26.0, 32.0)	29.0 (26.0, 32.0)	29.0 (27.0, 33.0)	0.607	29.0 (26.0, 32.0)	28.0 (26.0, 31.8)	0.195
	CARS scales, median (IQR)^a^	34.0 (32.0, 37.0)	34.0 (32.0, 37.0)	34.0 (32.0, 37.0)	0.580	34.0 (32.0, 37.0)	34.0 (31.3, 36.0)	0.339
	< 30.0 scores, *n* (%)	49 (3.8%)	42 (4.0%)	7 (2.9%)	—	40 (3.5%)	9 (6.5%)	—
	30.0 ~ 36.0 scores, *n* (%)	877 (68.6%)	717 (68.9%)	160 (66.9%)		782 (68.5%)	95 (68.8%)	
	> 36.0 scores, *n* (%)	353 (27.6%)	281 (27.0%)	72 (30.1%)		319 (28.0%)	34 (24.6%)	
GDS scales (*n* = 1176)^b^	Adaptive behavior DQ, means ± SD	54.3 ± 14.4	54.5 ± 14.5	53.4 ± 14.0	—	54.7 ± 14.4	42.6 ± 11.0	—
	> 85.0 scores, *n* (%)	15 (1.3%)	12 (1.3%)	3 (1.3%)	—	15 (1.3%)	0 (0.0%)	—
	76.0 ~ 85.0 scores, *n* (%)	72 (6.1%)	60 (6.3%)	12 (5.3%)		72 (6.3%)	0 (0.0%)	
	55.0 ~ 75.0 scores, *n* (%)	488 (41.5%)	397 (41.8%)	91 (40.3%)		484 (42.4%)	4 (11.4%)	
	40.0 ~ 54.0 scores, *n* (%)	420 (35.7%)	336 (35.4%)	84 (37.2%)		403 (35.3%)	17 (48.6%)	
	25.0 ~ 39.0 scores, *n* (%)	160 (13.6%)	129 (13.6%)	31 (13.7%)		147 (12.9%)	13 (37.1%)	
	< 25.0 scores, *n* (%)	21 (1.8%)	16 (1.7%)	5 (2.2%)		20 (1.8%)	1 (2.9%)	
	Gross motor DQ, median (IQR)	65.0 (55.0, 75.0)	65.0 (56.0, 75.0)	66.0 (55.0, 75.0)	—	66.0 (56.0, 76.0)	52.0 (47.0, 59.5)	—
	> 85.0 scores, *n* (%)	106 (9.0%)	87 (9.2%)	19 (8.4%)	—	105 (9.2%)	1 (2.9%)	—
	76.0 ~ 85.0 scores, *n* (%)	182 (15.5%)	148 (15.6%)	34 (15.0%)		181 (15.9%)	1 (2.9%)	
	55.0 ~ 75.0 scores, *n* (%)	618 (52.6%)	498 (52.4%)	120 (53.1%)		606 (53.1%)	12 (34.3%)	
	40.0 ~ 54.0 scores, *n* (%)	243 (20.7%)	195 (20.5%)	48 (21.2%)		224 (19.6%)	19 (54.3%)	
	25.0 ~ 39.0 scores, *n* (%)	27 (2.3%)	22 (2.3%)	5 (2.2%)		25 (2.2%)	2 (5.7%)	
	< 25.0 scores, *n* (%)	0 (0.0%)	0 (0.0%)	0 (0.0%)		0 (0.0%)	0 (0.0%)	
	Fine motor DQ, median (IQR)	59.0 (47.0, 71.0)	59.0 (47.0, 71.8)	60.5 (47.0, 70.0)	—	60.0 (48.0, 71.0)	47.0 (42.5, 58.0)	—
	> 85.0 scores, *n* (%)	75 (6.4%)	60 (6.3%)	15 (6.6%)	—	75 (6.6%)	0 (0.0%)	—
	76.0 ~ 85.0 scores, *n* (%)	141 (12.0%)	117 (12.3%)	24 (10.6%)		139 (12.2%)	2 (5.7%)	
	55.0 ~ 75.0 scores, *n* (%)	509 (43.3%)	404 (42.5%)	105 (46.5%)		499 (43.7%)	10 (28.6%)	
	40.0 ~ 54.0 scores, *n* (%)	322 (27.4%)	265 (27.9%)	57 (25.2%)		305 (26.7%)	17 (48.6%)	
	25.0 ~ 39.0 scores, *n* (%)	116 (9.9%)	93 (9.8%)	23 (10.2%)		111 (9.7%)	5 (14.3%)	
	< 25.0 scores, *n* (%)	13 (1.1%)	11 (1.2%)	2 (0.9%)		12 (1.1%)	1 (2.9%)	
	Language DQ, median (IQR)	35.0 (24.0, 47.0)	35.0 (24.0, 47.0)	34.5 (24.0, 46.0)	—	35.0 (24.0, 47.0)	29.0 (23.0, 37.5)	—
	> 85.0 scores, *n* (%)	8 (0.7%)	7 (0.7%)	1 (0.4%)	—	8 (0.7%)	0 (0.0%)	—
	76.0 ~ 85.0 scores, *n* (%)	9 (0.8%)	8 (0.8%)	1 (0.4%)		9 (0.8%)	0 (0.0%)	
	55.0 ~ 75.0 scores, *n* (%)	143 (12.2%)	115 (12.1%)	28 (12.4%)		142 (12.4%)	1 (2.9%)	
	40.0 ~ 54.0 scores, *n* (%)	323 (27.5%)	265 (27.9%)	58 (25.7%)		318 (27.9%)	5 (14.3%)	
	25.0 ~ 39.0 scores, *n* (%)	388 (33.0%)	309 (32.5%)	79 (35.0%)		370 (32.4%)	18 (51.4%)	
	< 25.0 scores, *n* (%)	305 (25.9%)	246 (25.9%)	59 (26.1%)		294 (25.8%)	11 (31.4%)	
	Personal‐social behavior DQ, median (IQR)	48.0 (40.0, 56.0)	48.0 (40.0, 56.0)	48.5 (40.0, 58.0)	—	—	41.0 (33.5, 54.0)	—
	> 85.0 scores, *n* (%)	8 (0.7%)	6 (0.6%)	2 (0.9%)	—	8 (0.7%)	0 (0.0%)	—
	76.0 ~ 85.0 scores, *n* (%)	31 (2.6%)	22 (2.3%)	9 (4.0%)		31 (2.7%)	0 (0.0%)	
	55.0 ~ 75.0 scores, *n* (%)	311 (26.4%)	251 (26.4%)	60 (26.5%)		302 (26.5%)	9 (25.7%)	
	40.0 ~ 54.0 scores, *n* (%)	540 (45.9%)	437 (46.0%)	103 (45.6%)		531 (46.5%)	9 (25.7%)	
	25.0 ~ 39.0 scores, *n* (%)	259 (22.0%)	211 (22.2%)	48 (21.2%)		243 (21.3%)	16 (45.7%)	
	< 25.0 scores, *n* (%)	27 (2.3%)	23 (2.4%)	4 (1.8%)		26 (2.3%)	1 (2.9%)	
Wechsler scales (*n* = 250)^c^	Normal range	32 (12.8%)	25 (11.6%)	7 (20.0%)	—	16 (14.3%)	16 (11.6%)	—
	Borderline	31 (12.4%)	30 (14.0%)	1 (2.9%)	—	16 (14.3%)	15 (10.9%)	—
	Intellectual disability	187 (74.8%)	160 (74.4%)	27 (77.1%)	—	80 (71.4%)	107 (77.5%)	—

Abbreviations: CARS, Childhood Autism Rating Scale; DQ, developmental quotient; GDS, Gesell Developmental Schedule; IQR, interquartile ranges; SD, standard deviations.

^a^ Individuals with a total score below 30 were excluded from autism classification. Those with a total score above 36 and a score of 3 or higher on at least five of the 15 subscales were classified as having severe autism. The remaining scores were classified as mild‐to‐moderate. In this study, CARS was used as a measure of symptom severity rather than a diagnostic tool; participants with scores below 30 were still included if they met diagnostic criteria.

^b^ GDS generates individual DQ scores for each domain, normal (> 85.0), borderline (76.0 ~ 85.0), mild delay (55.0 ~ 75.0), moderate delay (40.0 ~ 54.0), severe delay (25.0 ~ 39.0) and extremely severe delay (< 25.0).

^c^ Wechsler scales (WPPSI, WISC‐III or WISC‐IV) full‐scale IQ, normal (> 80.0), borderline (70.0 ~ 79.0), intellectual disability (< 70.0).

### 3.2 Prevalence of Comorbidities

**FIGURE 2:**
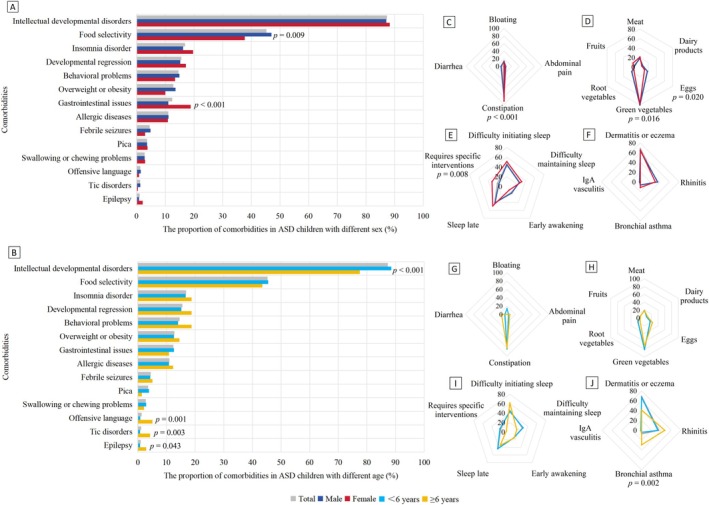
Proportion of comorbidities in children with ASD by sex and age groups. (A) Proportion of comorbidities in children with ASD by sex; (B) Proportion of comorbidities in children with ASD by age group; (C) Ratio of functional gastrointestinal issues in children with ASD by sex; (D) Proportion of foods rejected due to food selectivity in children with ASD by sex; (E) Proportion of common insomnia symptoms in children with ASD by sex; (F) Ratio of allergic diseases in children with ASD by sex; (G) Ratio of functional gastrointestinal issues in children with ASD by age group; (H) Proportion of foods rejected due to food selectivity in children with ASD by age group; (I) Proportion of common insomnia symptoms in children with ASD by age group; (J) Ratio of allergic diseases in children with ASD by age group.

Comorbidities were observed in 96.6% (*n* = 1236) of children with ASD, and 71.2% (*n* = 911) had two or more comorbid conditions. The three most prevalent were: IDD (87.3%), food selectivity (45.3%), and insomnia disorder (16.9%). Other notable comorbidities included: developmental regression (15.6%), behavioral problems (14.6%), overweight or obesity (12.8%), gastrointestinal issues (12.4%), allergic diseases (11%), febrile seizures (4.5%), pica (3.6%), swallowing or chewing difficulties (2.8%), offensive language (1.3%), tic disorders (1.2%), and epilepsy (1%). Details are provided in Table 2 and Figure 2. Additionally, among 29 intellectually normal children with ASD aged ≥ 6 years who underwent comprehensive Attention‐Deficit/Hyperactivity Disorder (ADHD) assessment, comorbid ADHD was identified in 24 (82.6%). In a subgroup of nine children aged ≥ 8 years assessed for anxiety and depression, 2 presented with both conditions, and one presented with anxiety symptoms alone. Owing to developmental constraints, structured psychiatric assessments were feasible in only small subgroups and thus were not included in the multivariable analyses.

**TABLE 2:** The *χ* ^2^ test results on the comorbidities of ASD children across different sex and age groups.

Comorbidities		Total (*n* = 1279)	Male (*n* = 1040)	Female (*n* = 239)	*p* (sex)	< 6 years (*n* = 1141)	≥ 6 years (*n* = 138)	*p* (age)
		*n* (%)	*n* (%)	*n* (%)		*n* (%)	*n* (%)	
Mental disorders	Intellectual developmental disorders	1117 (87.3%)	906 (87.1%)	211 (88.3%)	0.624	1010 (88.5%)	107 (77.5%)	< 0.001
	Insomnia disorder	216 (16.9%)	169 (16.2%)	47 (19.7%)	0.204	190 (16.7%)	26 (18.8%)	0.517
	Difficulty initiating sleep	99 (7.7%)	75 (7.2%)	24 (10.0%)	0.140	83 (7.3%)	16 (11.6%)	0.073
	Sleep late	92 (7.2%)	69 (6.6%)	23 (9.6%)	0.107	83 (7.3%)	9 (6.5%)	0.747
	Difficulty maintaining sleep	60 (4.7%)	45 (4.3%)	15 (6.3%)	0.199	56 (4.9%)	4 (2.9%)	0.292
	Requires specific interventions	44 (3.4%)	29 (2.8%)	15 (6.3%)	0.008	42 (3.7%)	2 (1.4%)	0.221
	Early awakening	32 (2.5%)	28 (2.7%)	4 (1.7%)	0.363	28 (2.5%)	4 (2.9%)	0.770
	Pica	46 (3.6%)	37 (3.6%)	9 (3.8%)	0.876	44 (3.9%)	2 (1.4%)	0.223
	Tic disorders	15 (1.2%)	14 (1.3%)	1 (0.4%)	0.329	9 (0.8%)	6 (4.3%)	0.003
Physical diseases	Overweight or obesity	164 (12.8%)	140 (13.5%)	24 (10.0%)	0.154	144 (12.6%)	20 (14.5%)	0.534
	Overweight	99 (7.7%)	85 (8.2%)	14 (5.9%)	0.227	87 (7.6%)	12 (8.7%)	0.657
	Obesity	65 (5.1%)	55 (5.3%)	10 (4.2%)	0.483	57 (5.0%)	8 (5.8%)	0.686
	Gastrointestinal issues	159 (12.4%)	114 (11.0%)	45 (18.8%)	< 0.001	144 (12.6%)	15 (10.9%)	0.556
	Functional constipation	131 (10.2%)	90 (8.7%)	41 (17.2%)	< 0.001	119 (10.4%)	12 (8.7%)	0.526
	Functional bloating	20 (1.6%)	15 (1.4%)	5 (2.1%)	0.401	20 (1.8%)	0 (0.0%)	0.156
	Functional diarrhea	9 (0.7%)	9 (0.9%)	0 (0.0%)	0.223	7 (0.6%)	2 (1.4%)	0.252
	Functional abdominal pain	6 (0.5%)	5 (0.5%)	1 (0.4%)	1.000	5 (0.4%)	1 (0.7%)	0.497
	Allergic diseases	141 (11.0%)	115 (11.1%)	26 (10.9%)	0.936	124 (10.9%)	17 (12.3%)	0.607
	Allergic dermatitis or eczema	90 (7.0%)	73 (7.0%)	17 (7.1%)	0.959	83 (7.3%)	7 (5.1%)	0.339
	Allergic rhinitis	49 (3.8%)	41 (3.9%)	8 (3.3%)	0.666	41 (3.6%)	8 (5.8%)	0.203
	Bronchial asthma	10 (0.8%)	7 (0.7%)	3 (1.3%)	0.408	5 (0.4%)	5 (3.6%)	0.002
	IgA vasculitis	2 (0.2%)	2 (0.2%)	0 (0.0%)	—	2 (0.2%)	0 (0.0%)	—
	Febrile seizures	57 (4.5%)	50 (4.8%)	7 (2.9%)	0.204	50 (4.4%)	7 (5.1%)	0.710
	Epilepsy	13 (1.0%)	8 (0.8%)	5 (2.1%)	0.077	9 (0.8%)	4 (2.9%)	0.043
Other problems	Food selectivity	579 (45.3%)	489 (47.0%)	90 (37.7%)	0.009	519 (45.5%)	60 (43.5%)	0.654
	Resistance to green vegetables	461 (36.0%)	391 (37.6%)	70 (29.3%)	0.016	418 (36.6%)	43 (31.2%)	0.206
	Resistance to root vegetables	110 (8.6%)	97 (9.3%)	13 (5.4%)	0.053	102 (8.9%)	8 (5.8%)	0.214
	Resistance to meat	110 (8.6%)	91 (8.8%)	19 (7.9%)	0.691	99 (8.7%)	11 (8.0%)	0.780
	Resistance to eggs	100 (7.8%)	90 (8.7%)	10 (4.2%)	0.020	86 (7.5%)	14 (10.1%)	0.281
	Resistance to fruits	67 (5.2%)	52 (5.0%)	15 (6.3%)	0.425	60 (5.3%)	7 (5.1%)	0.926
	Resistance to dairy products	36 (2.8%)	32 (3.1%)	4 (1.7%)	0.237	31 (2.7%)	5 (3.6%)	0.581
	Behavioral problems	187 (14.6%)	155 (14.9%)	32 (13.4%)	0.550	161 (14.1%)	26 (18.8%)	0.137
	Aggressive behavior	143 (11.2%)	118 (11.3%)	25 (10.5%)	0.695	122 (10.7%)	21 (15.2%)	0.111
	Self‐injurious behavior	68 (5.3%)	52 (5.0%)	16 (6.7%)	0.292	57 (5.0%)	11 (8.0%)	0.141
	Developmental regression	199 (15.6%)	158 (15.2%)	41 (17.2%)	0.450	173 (15.2%)	26 (18.8%)	0.260
	Swallowing or chewing problems	36 (2.8%)	29 (2.8%)	7 (2.9%)	0.906	33 (2.9%)	3 (2.2%)	0.790
	Offensive language	17 (1.3%)	15 (1.4%)	2 (0.8%)	0.753	10 (0.9%)	7 (5.1%)	0.001

*Note*: For comparisons involving categorical variables with expected cell counts < 5, Fisher's exact test was used instead of the *χ* ^2^ test, and the corresponding *p*‐values are reported. For conditions with extremely low overall frequency (e.g., IgA vasculitis, total *n* = 2), *p*‐values are not displayed due to insufficient sample size for meaningful comparison.

### 3.3 Comorbidities by Sex and Age Group

Gastrointestinal issues were significantly more common in girls (18.8%) than boys (11%) (*p* < 0.001), particularly functional constipation (17.2% vs. 8.7%, *p* < 0.001). Sleep‐related interventions (e.g., cuddling/light) were more frequently needed in girls (6.3% vs. 2.8%, *p* = 0.008). Compared to girls, boys exhibited significantly higher rates of both overall food selectivity (47% vs. 37.7%, *p* = 0.009) and the rejection of specific items, notably green vegetables (37.6% vs. 29.3%, *p* = 0.016) and eggs (8.7% vs. 4.2%, *p* = 0.020).

Children aged ≥ 6 years showed higher prevalence of: tic disorders (4.3% vs. 0.8%, *p* = 0.003); bronchial asthma (3.6% vs. 0.4%, *p* = 0.002); offensive language (5.1% vs. 0.9%, *p* = 0.001), and epilepsy (2.9% vs. 0.8%, *p* = 0.043). Children < 6 years had higher prevalence of IDD (88.5% vs. 77.5%, *p* < 0.001). However, these findings in older children should be interpreted with caution due to the small sample size (*n* = 138) and low case numbers for certain conditions (most cell values < 10 observations). More information can be found in Table 2 and Figure 2.

### 3.4 Association Between Comorbidities and Autism Severity (CARS)

**FIGURE 3:**
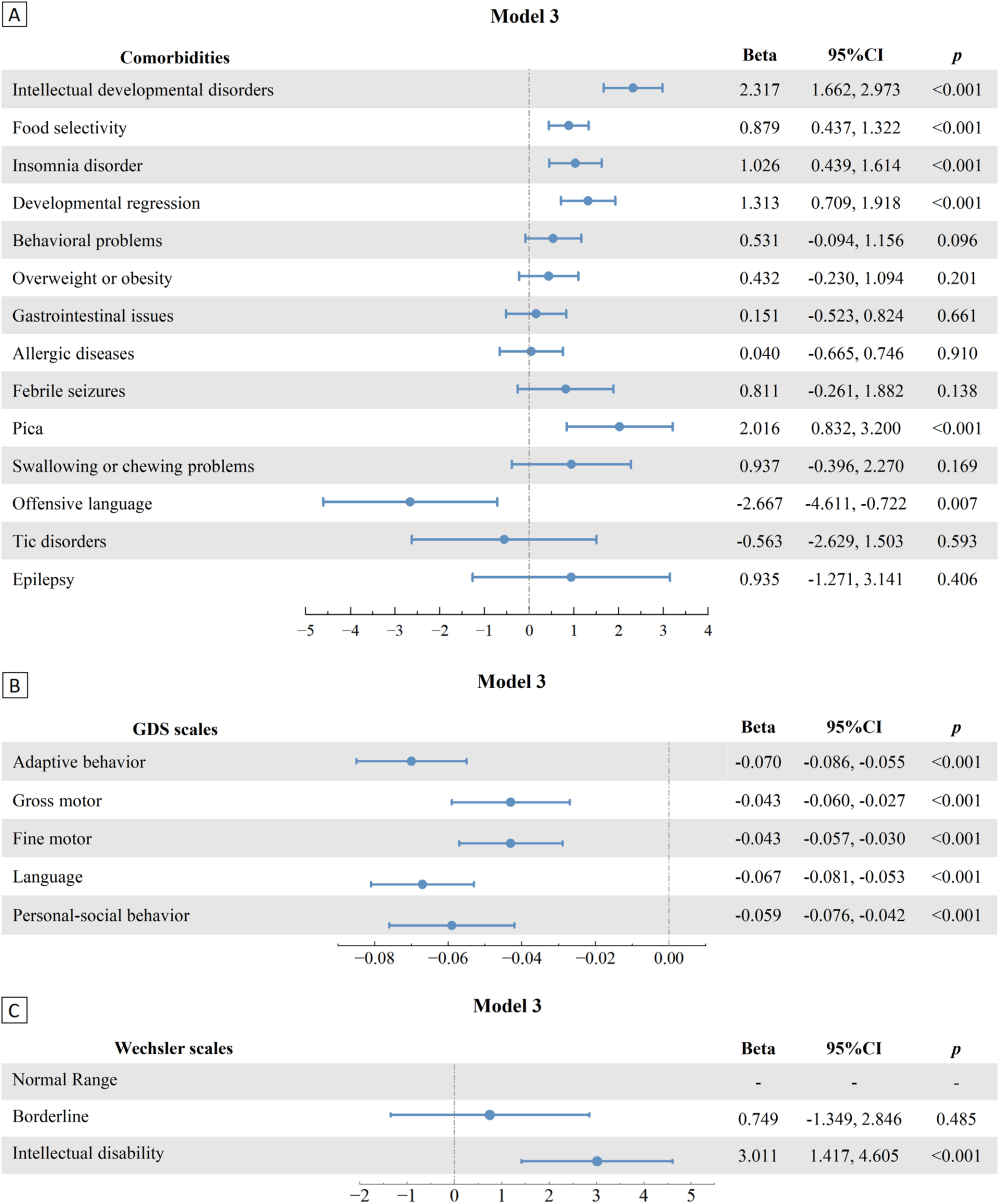
Generalized linear model 3 for the relationship between comorbidities, developmental status and CARS scores in Children with ASD. (A) Generalized linear Model 3 for comorbidities and CARS scores. (B) Generalized linear Model 3 for GDS and CARS scores. (C) Generalized linear Model 3 for Wechsler and CARS scores. CARS, Childhood Autism Rating Scale; GDS, Gesell Developmental Schedule.

Generalized linear regression models, adjusted for covariates, identified significant associations between CARS scores and specific comorbidities. Positive associations were found for IDD (*β* = 2.317, 95% CI: 1.662–2.973, *p* < 0.001), food selectivity (*β* = 0.879, 95% CI: 0.437–1.322, *p* < 0.001), insomnia disorder (*β* = 1.026, 95% CI: 0.439–1.614, *p* < 0.001), developmental regression (*β* = 1.313, 95% CI: 0.709–1.918, *p* < 0.001), and pica (*β* = 2.016, 95% CI: 0.832–3.200, *p* < 0.001). Conversely, offensive language (*β* = −2.667, 95% CI: −4.611 to −0.722, *p* = 0.007) was negatively correlated with CARS scores. IDD was associated with the largest increase in CARS scores among examined comorbidities. Full specifications of all statistical models are detailed in Figure 3 and Table S2.

### 3.5 Association Between Developmental Status and Autism Severity (CARS)

To assess the link between developmental status and autism severity, we conducted covariate‐adjusted generalized linear regression analyses. First, in models using GDS domains, all five GDS domains were significantly negatively associated with CARS scores: adaptive behavior (*β* = −0.070, 95% CI: −0.086 to −0.055, *p* < 0.001), gross motor (*β* = −0.043, 95% CI: −0.060 to −0.027, *p* < 0.001), fine motor (*β* = −0.043, 95% CI: −0.057 to −0.030, *p* < 0.001), language (*β* = −0.067, 95% CI: −0.081 to −0.053, *p* < 0.001), and personal‐social behavior (*β* = −0.059, 95% CI: −0.076 to −0.042, *p* < 0.001). Second, in models based on Wechsler scales, the presence of an ID was a strong positive predictor of CARS scores, associated with a mean increase of approximately three points (*β* = 3.011, 95% CI: 1.417–4.605, *p* < 0.001). Lower developmental scores were associated with higher CARS scores. Detailed specifications for all statistical models are presented in Figure 3 and Table S2.

## 4 Discussion

This multicenter clinical study provides a comprehensive evaluation of comorbidities in Chinese children with ASD based on clinician‐confirmed diagnoses. By examining data from 1279 children across eight geographically distributed medical centers, we present detailed estimates of comorbidity prevalence, age‐ and sex‐related patterns, and associations between comorbidities, developmental measures, and autism severity measured by CARS. To our knowledge, this is among the larger ASD cohorts in China assessed using standardized tools and multidisciplinary clinical evaluation.

We observed a substantial burden of comorbidity in this cohort, with 96.6% of children presenting with at least one comorbid condition and 71.2% presenting with multiple co‐occurring conditions. IDD was the most prevalent comorbidity (87.3%), followed by food selectivity (45.3%) and insomnia disorder (16.9%). Several comorbidities—IDD, food selectivity, pica, insomnia disorder, and developmental regression—were significantly associated with higher CARS scores, indicating greater autism symptom severity. Conversely, better developmental functioning across all GDS domains, particularly language and adaptive behavior, was associated with lower CARS scores, while a Wechsler full‐scale IQ below 70 was associated with greater symptom severity.

The IDD prevalence in our study is notably higher than in Western reports, where it ranges from 21.7% to 33% (Khachadourian et al. 2023; Micai et al. 2023). This discrepancy may reflect differences in diagnostic timing, referral patterns, and limited access to early screening services in China. Our cohort consisted primarily of children under 6 years old, and assessments were conducted in tertiary hospitals, which may have resulted in overrepresentation of more severe or complex cases. We also observed a decline in IDD rates in older children, possibly due to early intervention, neurodevelopmental maturation, or delayed diagnosis in milder cases. These findings are consistent with prior reports that intellectual impairment is associated with greater ASD symptom burden (Matson and Shoemaker 2009). The developmental profile of children with ASD and GDD revealed asymmetrical delays, with gross motor skills better preserved than language and personal‐social skills—distinct from children with GDD alone (Shan et al. 2021).

Our epilepsy rate (1%) and tic disorder rate (1.2%) are lower than commonly reported (16% and 10%) in Western adolescent samples (Micai et al. 2023). This discrepancy likely reflects the younger age of our cohort and potential underdiagnosis due to variable access to pediatric neurologists across sites. In addition, acute epilepsy or seizure cases were referred to specialized departments and excluded if they lacked stable behavioral assessments. The prevalence of overweight (12.8%) and gastrointestinal disorders (12.4%) was also lower than reported in Western studies (Healy et al. 2019; Micai et al. 2023), potentially due to cultural dietary habits or our emphasis on more severe clinical symptoms (Hong et al. 2023; Wong and Srivastava 2023).

Sex‐based differences were observed, with girls exhibiting higher rates of gastrointestinal symptoms and sleep‐related interventions, and boys demonstrating higher rates of food selectivity. Although biological explanations have been proposed in prior literature (Sotelo‐Orozco and Hertz‐Picciotto 2025; Wallace et al. 2021), our cross‐sectional data do not allow inference regarding underlying mechanisms. These findings should therefore be interpreted descriptively. The higher male‐to‐female ratio with ASD (4.4:1) observed is consistent with the general epidemiology of ASD (3.8:1) (Maenner et al. 2023).

Older children (≥ 6 years) demonstrated higher rates of tic disorders, asthma, epilepsy, and offensive language. Tic disorders are known to peak during school‐age years (Singer 2019), which may partially explain this pattern. However, because our study design is cross‐sectional, these differences should not be interpreted as reflecting developmental progression. Longitudinal studies are needed to clarify whether specific comorbidities change over time.

We identified a cluster of comorbidities—IDD, food selectivity, pica, insomnia, and developmental regression—that were associated with higher CARS scores. These co‐occurring conditions may reflect increased clinical complexity in children presenting with greater symptom severity. However, the cross‐sectional design precludes conclusions regarding directionality or mechanistic pathways.

A key observation is the partial discrepancy between the post‐imputation and complete‐case results. This underscores the capacity of multiple imputation to enhance statistical power and mitigate selection bias. Notably, the comorbidity‐severity associations observed in the younger ASD group (< 6 years, *n* = 1141) mirrored those in the imputed dataset. However, in the older subgroup (≥ 6 years, *n* = 138), only IDD and food selectivity remained significantly associated with disease severity. The loss of other associations may reflect insufficient statistical power due to the substantially smaller sample size of the older cohort. Importantly, both methods confirmed the stable link between key comorbidities (e.g., IDD, developmental regression, food selectivity) and ASD severity. Although minor differences were observed between imputed and complete‐case analyses, the direction and magnitude of key associations remained stable. These findings support the robustness of the results while acknowledging the assumptions inherent in multiple imputation procedures.

## 5 Limitations and Future Directions

A major strength of this study is its large sample size, geographic diversity, and reliance on multidisciplinary, clinician‐confirmed diagnoses. The use of validated tools (CARS, GDS, and Wechsler scales) adds robustness to our assessment of ASD severity and developmental status. However, several limitations must be acknowledged. First, as participants were recruited exclusively from tertiary hospitals, our sample may not be fully representative of the general population of children with ASD, potentially introducing a selection bias toward those with more pronounced symptom severity or complex clinical needs. Second, the underrepresentation of children aged ≥ 6 years in our cohort resulted in limited statistical power, and the number of detected cases for certain comorbid conditions was low. Further validation in larger cohorts with a more balanced age distribution is warranted to replicate these findings. Third, while the exclusion of children with acute conditions such as active epileptic seizures was necessary for safety and procedural consistency, this may limit the generalizability of our findings to the full phenotypic heterogeneity of ASD. Finally, the assessment of psychiatric comorbidities (e.g., ADHD, anxiety) was constrained by the cognitive and communicative capacities of some participants, preventing more in‐depth profiling. Furthermore, the significant overlap of psychiatric symptoms made it difficult to determine, given these communication limitations, whether functional impairments were primary to the presenting illness or secondary to other comorbid conditions. Future research should prioritize community‐based recruitment, longitudinal designs, and developmentally adapted assessments to better capture evolving comorbidity patterns and developmental trajectories across the lifespan.

## 6 Conclusions

In this large multicenter clinical cohort, children with ASD demonstrated a high prevalence of comorbid conditions, with distinct patterns across sex and age groups. IDD, food selectivity, pica, insomnia disorder, and developmental regression were associated with higher CARS scores, while stronger developmental performance was associated with lower severity scores. These findings underscore the importance of comprehensive developmental and medical evaluation in children with ASD and contribute evidence from an underrepresented population.

## Author Contributions

Changlian Zhu had full access to all the data in the study and takes responsibility for the integrity and accuracy of the data and analysis. Concept and design: Changlian Zhu, Dizhou Pang, Yiran Xu. Data acquisition, analysis, or interpretation: Dizhou Pang, Guiqin Duan, Qing Shang, Huichun Zhang, Binbin Wang, Huaili Ding, Huihui Kang, Yue Zhao, Yuxia Lu, Gai Tian, Li Zhang, Wu Jiang, Ganyu Wang, Cailing Liang, Zhenghua Li, Chunlan Song, Yanyan Yang, Yazhe Wang, Kaichen Yue, Yongbin Lang, Jiatian Liu, Changlian Zhu. Manuscript drafting: Dizhou Pang, Changlian Zhu. Critical review for important intellectual content: Yiran Xu, Lingling Zhang, Bingbing Li, Xiaoli Zhang. Statistical analysis: Dizhou Pang, Changlian Zhu, Lingling Zhang, Bingbing Li, Xiaoli Zhang. Funding acquisition: Changlian Zhu, Dizhou Pang. Administrative, technical, or material support: Changlian Zhu. Supervision: Changlian Zhu.

## Funding

This study was supported by grant U21A20347 from the National Natural Science Foundation of China, grant SBGJ202301009 from the Henan Medical Science Foundation, grants GZS2023003 and 241111521300 from the Henan Science and Technology Foundation, grant 2022‐01019 from the Swedish Research Council, the Adlerbert Research Foundation (2023‐684, 2024‐822), Stiftelsen Edit Jacobsons Donationsfond (2024‐150), grant KFKT2021006 from the Joint Open Research Fund of Henan Key Laboratory of Child Brain Injury and Henan Pediatric Clinical Research Center.

## Ethics Statement

This research was approved by the Medical Ethics Committee of the Third Affiliated Hospital of Zhengzhou University (Approval No. 2022‐021‐01). Under the multicenter ethics governance arrangement for this study, all participating centers accepted the lead‐center approval and completed local administrative filing procedures before participant enrollment. Written informed consent was obtained from the legal guardians of all participants prior to study participation. A physician explained the study procedures and answered any questions from the children and their guardians. Participation was voluntary, and participants and their guardians were informed of their right to withdraw at any time without consequence. Age‐appropriate assent was obtained from children whenever feasible. All data were handled confidentially. This study involved no therapeutic intervention.

## Conflicts of Interest

The authors declare no conflicts of interest.

## Supporting information


**Table S1:** Supporting Information.

**Table S2:** Supporting Information.

**Table S3:** Supporting Information.

**Table S4:** Supporting Information.

**Table S5:** Supporting Information.

**Table S6:** Supporting Information.

**Table S7:** Supporting Information.

## Data Availability

The data that support the findings of this study are available on request from the corresponding author. The data are not publicly available due to privacy or ethical restrictions.
