# Supplementary material for: Associations Between Comorbidities, Developmental Status, and Disease Severity in Children With Autism Spectrum Disorder: A Multicenter Cross‐Sectional Study in China
Source: Autism Res. 2026 Apr 13;19(6):e70253. doi: 10.1002/aur.70253 (PMC13276685; doi:10.1002/aur.70253)
Supplement: Supplementary file 1 — Table S1: Supporting Information. [file AUR-19-0-s005.docx]

| **Comorbidity category**  ****Table S1: The standardized assessment protocol for comorbidities**** | **Specific condition** | **Primary diagnostic standard / tool** | **Assessor (minimum qualification)** |
| --- | --- | --- | --- |
| **Autism spectrum disorder** | - | **DSM-5 criteria & scale assessment (e.g., CARS, ADOS, ABC, CHAT)** | **Developmental-behavioral pediatrician / psychiatrist** |
| **Intellectual developmental disorders** | **Intellectual disability (ID)** | **DSM-5 criteria & Wechsler IQ < 70 (WPPSI, WISC)** | **Developmental-behavioral pediatrician / psychiatrist** |
|  | **Global developmental delay (GDD)** | **DSM-5 criteria & GDS DQ ≤ 75 in ≥2 domains** |  |
| **Insomnia disorder** | **Difficulty initiating sleep, etc.** | **ICD-10 criteria & caregiver questionnaire（e.g., CSHQ, ASHS）** | **Developmental-behavioral pediatrician / psychiatrist** |
| **Gastrointestinal issues** | **Functional constipation, etc.** | **Clinical evaluation(e.g., abdominal ultrasound) & Rome IV criteria** | **Pediatrician / gastroenterologist** |
| **Nutritional** | **Overweight/obesity** | **WHO BMI-for-age z-score** | **Developmental-behavioral pediatrician / pediatrician** |
| **Neurological** | **Epilepsy** | **Clinical history & EEG findings (ILAE criteria)** | **Pediatric neurologist** |
|  | **Febrile seizures** | **AAP Guidelines (2011)** | **Pediatric neurologist / pediatrician** |
| **Behavioral** | **Behavioral problems** | **Caregiver interview & direct behavioral observation (CBCL, documented frequency and impact) ^A^** | **Developmental-behavioral pediatrician / psychiatrist** |
|  | **Developmental regression** | **Clinical history (loss of skills > 3 months)** |  |
|  | Offensive language | **Direct behavioral observation & caregiver interview (documented frequency and context)** |  |
| **Allergic/immune** | **Asthma, allergic rhinitis, etc.** | **Clinical history & objective tests (e.g., lung function, allergen test)** | **Pediatric allergist / pulmonologist** |
| **Feeding/eating** | **Food selectivity** | **Detailed caregiver interview & food log review** | **Developmental-behavioral pediatrician / psychiatrist / physiatrist** |
|  | **Pica** | **DSM-5 criteria** |  |
|  | Swallowing or chewing problems | **Clinical feeding evaluation & caregiver report (based on functional impact)** |  |
| Other mental disorders | ADHD | DSM-5 criteria & scale assessment (e.g., WFIRS,SNAP-IV, ) | **Developmental-behavioral pediatrician / psychiatrist** |
|  | Anxiety symptoms | DSM-5 criteria & scale assessment (e.g., CBCL, SAS) |  |
|  | Depressive symptoms | DSM-5 criteria & scale assessment (e.g., CBCL, SDS) |  |

^A^ Positive when one or more challenging behaviors were repeatedly present and judged clinically significant because they caused functional impairment, safety concerns, or required specific management/intervention.

Abbreviations: DSM-5, Diagnostic and Statistical Manual of Mental Disorders, Fifth Edition; CARS, The Childhood Autism Rating Scale; ADOS, Autism Diagnostic Observation Schedule; ABC, The Autism Behavior Checklist; CHAT, The Checklist for Autism in Toddlers; ID, Intellectual disability; GDD, Global developmental delay; IQ, intelligence quotient; DQ, Developmental quotient; WPPSI, The Wechsler Preschool and Primary Scale of Intelligence; WISC, The Wechsler Intelligence Scale for Children; GDS, The Gesell Developmental Schedule; ICD-10, The International Statistical Classification of Diseases and Related Health Problems 10th Revision; CSHQ, The Children's Sleep Health Questionnaire; ASHS, The Adolescent Sleep Hygiene Scale; **EEG, Electroencephalography;** ILAE, International League Against Epilepsy; AAP Guidelines, Febrile Seizures: Guideline for the Neurodiagnostic Evaluation of the Child With a Simple Febrile Seizure; WFIRS, Weiss Functional Im-pairment Rating Scales; SNAP-IV, Swanson,Nolan,and Pelham-IV rating scales; CBCL, Child Behavior Checklist; SAS, Self-rating Anxiety Scale; SDS, Self-Rating Depression Scale.
