# Supplementary material for: Associations Between Comorbidities, Developmental Status, and Disease Severity in Children With Autism Spectrum Disorder: A Multicenter Cross‐Sectional Study in China
Source: Autism Res. 2026 Apr 13;19(6):e70253. doi: 10.1002/aur.70253 (PMC13276685; doi:10.1002/aur.70253)
Supplement: Supplementary file 2 — Table S2: Supporting Information. [file AUR-19-0-s003.docx]

| Comorbidities  (n=1279) | CARS | | | | | | | | | |
| --- | --- | --- | --- | --- | --- | --- | --- | --- | --- | --- |
|  | Model 1 | | | | Model 2 | | | Model 3 | | |
|  | Beta | | 95%CI | *p* | Beta | 95%CI | *p* | Beta | 95%CI | *p* |
| IDD | 2.351 | | 1.699, 3.003 | <0.001 | 2.366 | 1.711, 3.022 | <0.001 | 2.317 | 1.662, 2.973 | <0.001 |
| Food selectivity | 0.860 | | 0.418, 1.301 | <0.001 | 0.878 | 0.435, 1.321 | <0.001 | 0.879 | 0.437, 1.322 | <0.001 |
| Insomnia disorder | 1.019 | | 0.432, 1.606 | <0.001 | 1.011 | 0.423, 1.600 | <0.001 | 1.026 | 0.439, 1.614 | <0.001 |
| Developmental regression | 1.333 | | 0.728, 1.938 | <0.001 | 1.328 | 0.722, 1.934 | <0.001 | 1.313 | 0.709, 1.918 | <0.001 |
| Behavior problems | 0.520 | | -0.104, 1.145 | 0.103 | 0.528 | -0.098, 1.154 | 0.099 | 0.531 | -0.094, 1.156 | 0.096 |
| Overweight or obesity | 0.444 | | -0.216, 1.105 | 0.188 | 0.457 | -0.204, 1.119 | 0.176 | 0.432 | -0.230, 1.094 | 0.201 |
| Gastrointestinal issues | 0.187 | | -0.483, 0.857 | 0.584 | 0.162 | -0.511, 0.836 | 0.637 | 0.151 | -0.523, 0.824 | 0.661 |
| Allergic diseases | -0.005 | | -0.710, 0.701 | 0.989 | -0.004 | -0.710, 0.702 | 0.991 | 0.040 | -0.665, 0.746 | 0.910 |
| Febrile seizures | 0.722 | | -0.348, 1.792 | 0.186 | 0.744 | -0.329, 1.816 | 0.175 | 0.811 | -0.261, 1.882 | 0.138 |
| Pica | 2.090 | | 0.909, 3.271 | <0.001 | 2.087 | 0.905, 3.270 | <0.001 | 2.016 | 0.832, 3.200 | <0.001 |
| Swallowing or chewing problems | 0.924 | | -0.412, 2.259 | 0.175 | 0.921 | -0.415, 2.257 | 0.177 | 0.937 | -0.396, 2.270 | 0.169 |
| Offensive language | -2.631 | | -4.555, -0.706 | 0.007 | -2.659 | -4.606, -0.711 | 0.008 | -2.667 | -4.611, -0.722 | 0.007 |
| Tic disorders | -0.587 | | -2.639, 1.465 | 0.575 | -0.554 | -2.616, 1.508 | 0.599 | -0.563 | -2.629, 1.503 | 0.593 |
| Epilepsy | 1.067 | | -1.136, 3.269 | 0.343 | 1.032 | -1.178, 3.242 | 0.360 | 0.935 | -1.271, 3.141 | 0.406 |
| GDS scales  (n=1176) | Model 1 | | | | Model 2 | | | Model 3 | | |
|  | Beta | 95%CI | | *p* | Beta | 95%CI | *p* | Beta | 95%CI | *p* |
| Adaptive behavior | -0.077 | -0.092, -0.061 | | <0.001 | -0.075 | -0.090, -0.059 | <0.001 | -0.070 | -0.086, -0.055 | <0.001 |
| Gross motor | -0.050 | -0.065, -0.034 | | <0.001 | -0.046 | -0.062, -0.029 | <0.001 | -0.043 | -0.060, -0.027 | <0.001 |
| Fine motor | -0.048 | -0.062, -0.034 | | <0.001 | -0.046 | -0.060, -0.032 | <0.001 | -0.043 | -0.057, -0.030 | <0.001 |
| Language | -0.076 | -0.090, -0.062 | | <0.001 | -0.074 | -0.088, -0.060 | <0.001 | -0.067 | -0.081, -0.053 | <0.001 |
| Personal-social behavior | -0.069 | -0.086, -0.052 | | <0.001 | -0.066 | -0.083, -0.049 | <0.001 | -0.059 | -0.076, -0.042 | <0.001 |
| Wechsler scales  (n=250) | Model 1 | | | | Model 2 | | | Model 3 | | |
|  | Beta | 95%CI | | *p* | Beta | 95%CI | *p* | Beta | 95%CI | *p* |
| Normal Range | - | - | | *-* | - | - | *-* | - | - | *-* |
| Borderline | 0.742 | -1.326, 2.810 | | 0.482 | 0.480 | -1.596, 2.555 | 0.651 | 0.749 | -1.349, 2.846 | 0.485 |
| Intellectual disability | 3.292 | 1.722, 4.862 | | <0.001 | 3.230 | 1.663, 4.798 | <0.001 | 3.011 | 1.417, 4.605 | <0.001 |

**Table S2 All generalized linear models for the relationship between comorbidities, developmental status and CARS scores in ASD children**

For the comorbidities–CARS models, Model 1 was unadjusted. Model 2 was adjusted for sex and age. Model 3 was additionally adjusted for premature birth, paternal age at conception, family history of mental illness, and gestational hypertension.

For the GDS–CARS and Wechsler–CARS models, Model 1 was unadjusted. Model 2 was adjusted for sex, age, premature birth, paternal age at conception, family history of mental illness, and gestational hypertension. Model 3 was further adjusted for all covariates in Model 2, plus food selectivity, developmental regression, offensive language, pica, and insomnia disorder.

Abbreviations: CARS, Childhood Autism Rating Scale; GDS, Gesell Developmental Schedule.
