# Supplementary material for: Associations Between Comorbidities, Developmental Status, and Disease Severity in Children With Autism Spectrum Disorder: A Multicenter Cross‐Sectional Study in China
Source: Autism Res. 2026 Apr 13;19(6):e70253. doi: 10.1002/aur.70253 (PMC13276685; doi:10.1002/aur.70253)
Supplement: Supplementary file 3 — Table S3: Supporting Information. [file AUR-19-0-s007.docx]

**Table S3 General characteristics of the participants in the study (complete case analysis)**

|  | | | | Total | Sex | | *p* values | Age | | *p* values |
| --- | --- | --- | --- | --- | --- | --- | --- | --- | --- | --- |
|  |  |  |  |  | Male | Female |  | < 6 years | ≥ 6 years |  |
| Basic information  (n=976) | Number of cases, n(%) | | | 976 (100.0%) | 797 (81.7%) | 179 (18.3%) | - | 868 (89.9%) | 108 (11.1%) | - |
|  | Age, median (IQR) | | | 4.2 (3.4, 5.1) | 4.2 (3.4, 5.2) | 4.0 (3.4, 4.8) | 0.108 | 4.0 (3.3, 4.8) | 6.7 (6.2, 7.0) | - |
|  | Premature birth, n(%) | | | 76 (7.8%) | 65 (8.2%) | 11 (6.1%) | 0.364 | 66 (7.6%) | 10 (9.3%) | 0.545 |
|  | Gestational hypertension, n(%) | | | 45 (4.6%) | 37 (4.6%) | 8 (4.5%) | 0.920 | 44 (5.1%) | 1 (0.9%) | 0.051 |
|  | Family history of mental illness, n(%) | | | 20 (2.0%) | 16 (2.0%) | 4 (2.2%) | 0.774 | 15 (1.7%) | 5 (4.6%) | 0.060 |
|  | Paternal age at conception, median (IQR) | | | 29.0 (26.0, 32.0) | 29.0 (26.0, 32.0) | 29.0 (27.0, 33.0) | 0.638 | 29.0 (26.0, 33.0) | 28.0 (25.0, 31.0) | 0.070 |
| ^A^ CARS  scales  (n=762) | CARS, median (IQR) | | | 34.0 (32.0, 37.0) | 34.0 (32.0, 37.0) | 34.0 (32.0, 37.0) | - | 34.5 (32.0, 37.0) | 33.0 (31.0, 36.0) | - |
|  |  | | < 30.0 scores, n(%) | 28 (3.7%) | 23 (3.7%) | 5 (3.7%) | - | 22 (3.2%) | 6 (7.6%) | - |
|  |  | | 30.0 ~ 36.0 scores, n(%) | 523 (68.6%) | 434 (69.2%) | 89 (65.9%) |  | 468 (68.5%) | 55 (69.6%) |  |
|  |  | | > 36.0 scores, n(%) | 211 (27.7%) | 170 (27.1%) | 41 (30.4%) |  | 193 (28.3%) | 18 (22.8%) |  |
| ^B^ GDS  scales  (n=607) | Adaptive behavior DQ, median (IQR) | | | 54.0 (44.0, 64.0) | 54.0 (44.0, 64.0) | 54.0 (45.3, 62.0) | - | 54.0 (44.0, 64.0) | 41.0 (35.0, 45.8) | - |
|  |  | > 85.0 scores, n(%) | | 11 (1.8%) | 9 (1.8%) | 2 (1.8%) | - | 11 (1.9%) | 0 (0.0%) | - |
|  |  | 76.0 ~ 85.0 scores, n(%) | | 45 (7.4%) | 35 (7.1%) | 10 (8.8%) |  | 45 (7.7%) | 0 (0.0%) |  |
|  |  | 55.0 ~ 75.0 scores, n(%) | | 237 (39.0%) | 195 (39.6%) | 42 (36.8%) |  | 235 (40.0%) | 2 (10.0%) |  |
|  |  | 40.0 ~ 54.0 scores, n(%) | | 215 (35.4%) | 171 (34.7%) | 44 (38.6%) |  | 206 (35.1%) | 9 (45.0%) |  |
|  |  | 25.0 ~ 39.0 scores, n(%) | | 84 (13.8%) | 72 (14.6%) | 12 (10.5%) |  | 76 (12.9%) | 8 (40.0%) |  |
|  |  | < 25.0 scores, n(%) | | 15 (2.5%) | 11 (2.2%) | 4 (3.5%) |  | 14 (2.4%) | 1 (5.0%) |  |
|  | Gross motor DQ, median (IQR) | | | 66.0 (57.0, 75.0) | 66.0 (57.0,75.0) | 69.0 (57.0, 75.8) | - | 67.0 (57.0, 76.0) | 52.5 (47.0, 60.5) | - |
|  |  | > 85.0 scores, n(%) | | 59 (9.7%) | 47 (9.5%) | 12 (10.5%) | - | 59 (10.1%) | 0 (0.0%) | - |
|  |  | 76.0 ~ 85.0 scores, n(%) | | 93 (15.3%) | 76 (15.4%) | 17 (14.9%) |  | 92 (15.7%) | 1 (5.0%) |  |
|  |  | 55.0 ~ 75.0 scores, n(%) | | 321 (52.9%) | 259 (52.5%) | 62 (54.4%) |  | 314 (53.5%) | 7 (35.0%) |  |
|  |  | 40.0 ~ 54.0 scores, n(%) | | 124 (20.4%) | 103 (20.9%) | 21 (18.4%) |  | 112 (19.1%) | 12 (60.0%) |  |
|  |  | 25.0 ~ 39.0 scores, n(%) | | 10 (1.6%) | 8 (1.6%) | 2 (1.8%) |  | 10 (1.7%) | 0 (0.0%) |  |
|  |  | < 25.0 scores, n(%) | | 0 (0.0%) | 0 (0.0%) | 0 (0.0%) |  | 0 (0.0%) | 0 (0.0%) |  |
|  | Fine motor DQ, median (IQR) | | | 59.0 (47.0, 71.0) | 59.0 (47.0,71.0) | 61.5 (46.0, 72.0) | - | 60.0 (48.0, 72.0) | 46.5 (40.5, 55.8) | - |
|  |  | > 85.0 scores, n(%) | | 46 (7.6%) | 36 (7.3%) | 10 (8.8%) | - | 46 (7.8%) | 0 (0.0%) | - |
|  |  | 76.0 ~ 85.0 scores, n(%) | | 76 (12.5%) | 62 (12.6%) | 14 (12.3%) |  | 76 (12.9%) | 0 (0.0%) |  |
|  |  | 55.0 ~ 75.0 scores, n(%) | | 251 (41.4%) | 201 (40.8%) | 50 (43.9%) |  | 245 (41.7%) | 6 (30.0%) |  |
|  |  | 40.0 ~ 54.0 scores, n(%) | | 161 (26.5%) | 132 (26.8%) | 29 (25.4%) |  | 152 (25.9%) | 9 (45.0%) |  |
|  |  | 25.0 ~ 39.0 scores, n(%) | | 65 (10.7%) | 55 (11.2%) | 10 (8.8%) |  | 61 (10.4%) | 4 (20.0%) |  |
|  |  | < 25.0 scores, n(%) | | 8 (1.3%) | 7 (1.4%) | 1 (0.9%) |  | 7 (1.2%) | 1 (5.0%) |  |
|  | Language DQ, median (IQR) | | | 34.0 (23.0, 47.0) | 34.0 (23.0,47.0) | 36.0 (24.3, 46.0) | - | 35.0 (23.0, 47.0) | 28.0 (22.3, 36.0) | - |
|  |  | > 85.0 scores, n(%) | | 5 (0.8%) | 4 (0.8%) | 1 (0.9%) | - | 5 (0.9%) | 0 (0.0%) | - |
|  |  | 76.0 ~ 85.0 scores, n(%) | | 7 (1.2%) | 6 (1.2%) | 1 (0.9%) |  | 7 (1.2%) | 0 (0.0%) |  |
|  |  | 55.0 ~ 75.0 scores, n(%) | | 73 (12.0%) | 62 (12.6%) | 11 (9.6%) |  | 72 (12.3%) | 1 (5.0%) |  |
|  |  | 40.0 ~ 54.0 scores, n(%) | | 156 (25.7%) | 121 (24.5%) | 35 (30.7%) |  | 155 (26.4%) | 1 (5.0%) |  |
|  |  | 25.0 ~ 39.0 scores, n(%) | | 200 (32.9%) | 163 (33.1%) | 37 (32.5%) |  | 188 (32.0%) | 12 (60.0%) |  |
|  |  | < 25.0 scores, n(%) | | 166 (27.3%) | 137 (27.8%) | 29 (25.4%) |  | 160 (27.3%) | 6 (30.0%) |  |
|  | Personal-social behavior DQ, median (IQR) | | | 48.0 (41.0, 57.0) | 48.0 (41.0,57.0) | 49.5 (42.3, 58.8) | - | 49.0 (41.0, 57.0) | 36.0 (32.8, 48.0) | - |
|  |  | > 85.0 scores, n(%) | | 5 (0.8%) | 4 (0.8%) | 1 (0.9%) | - | 5 (0.9%) | 0 (0.0%) | - |
|  |  | 76.0 ~ 85.0 scores, n(%) | | 14 (2.3%) | 9 (1.8%) | 5 (4.4%) |  | 14 (2.4%) | 0 (0.0%) |  |
|  |  | 55.0 ~ 75.0 scores, n(%) | | 170 (28.0%) | 139 (28.2%) | 31 (27.2%) |  | 166 (28.3%) | 4 (20.0%) |  |
|  |  | 40.0 ~ 54.0 scores, n(%) | | 291 (47.9%) | 234 (47.5%) | 57 (50.0%) |  | 287 (48.9%) | 4 (20.0%) |  |
|  |  | 25.0 ~ 39.0 scores, n(%) | | 114 (18.8%) | 96 (19.5%) | 18 (15.8%) |  | 103 (17.5%) | 11 (55.0%) |  |
|  |  | < 25.0 scores, n(%) | | 13 (2.1%) | 11 (2.2%) | 2 (1.8%) |  | 12 (2.0%) | 1 (5.0%) |  |
| ^C^ Wechsler scales  (n=116) | Normal Range | | | 13 (11.2%) | 11 (10.7%) | 2 (15.4%) | - | 6 (10.9%) | 7 (11.5%) | - |
|  | Borderline | | | 12 (10.3%) | 12 (11.7%) | 0 (0.0%) |  | 4 (7.3%) | 8 (13.1%) |  |
|  | Intellectual disability | | | 91 (78.4%) | 80 (77.7%) | 11 (84.6%) |  | 45 (81.8%) | 46 (75.4%) |  |

Of the 769 children with ASD who completed the GDS, 607 also had CARS data. A total of 163 children were assessed with the Wechsler scale, 116 of whom had concurrent CARS results. The GDS and Wechsler data presented are those included in the generalized linear regression analysis.

^A^ Individuals with a total score below 30 were excluded from autism classification. Those with a total score above 36 and a score of 3 or higher on at least five of the 15 subscales were classified as having severe autism. The remaining scores were classified as mild-to-moderate. In this study, CARS was used as a measure of symptom severity rather than a diagnostic tool; participants with scores below 30 were still included if they met diagnostic criteria.

^B^ GDS generates individual DQ scores for each domain, normal (> 85.0), borderline (76.0 ~ 85.0), mild delay (55.0 ~ 75.0), moderate delay (40.0 ~ 54.0), severe delay (25.0 ~ 39.0) and extremely severe delay (< 25.0).

^C^ Wechsler scales (WPPSI, WISC-III or WISC-IV) full-scale IQ, normal (> 80.0), borderline (70.0 ~ 79.0), intellectual disability (< 70.0).

Abbreviations: IQR, interquartile ranges; CARS, Childhood Autism Rating Scale; GDS, Gesell Developmental Schedule; DQ, developmental quotient.
