# Supplementary material for: Associations Between Comorbidities, Developmental Status, and Disease Severity in Children With Autism Spectrum Disorder: A Multicenter Cross‐Sectional Study in China
Source: Autism Res. 2026 Apr 13;19(6):e70253. doi: 10.1002/aur.70253 (PMC13276685; doi:10.1002/aur.70253)
Supplement: Supplementary file 4 — Table S4: Supporting Information. [file AUR-19-0-s006.docx]

**Table S4 The Chi-square test results on the comorbidities of ASD children across different sex and age groups (complete case analysis)**

| Comorbidities | | | Total (n=976) | Male (n=797) | Female (n=179) | *p* values  (sex) | < 6 years (n=868) | ≥ 6 years (n=108) | *p* values  (age) |
| --- | --- | --- | --- | --- | --- | --- | --- | --- | --- |
|  |  |  | n(%) | n(%) | n(%) |  | n(%) | n(%) |  |
| Mental disorders | Intellectual developmental disorders | | 858 (87.9%) | 700 (87.8%) | 158 (88.3%) | 0.871 | 775 (89.3%) | 83 (76.9%) | <0.001 |
|  | Insomnia disorder | | 164 (16.8%) | 130 (16.3%) | 34 (19.0%) | 0.386 | 145 (16.7%) | 19 (17.6%) | 0.816 |
|  |  | Difficulty initiating sleep | 77 (7.9%) | 58 (7.3%) | 19 (10.6%) | 0.134 | 64 (7.4%) | 13 (12.0%) | 0.090 |
|  |  | Sleep late | 74 (7.6%) | 56 (7.0%) | 18 (10.1%) | 0.166 | 67 (7.7%) | 7 (6.5%) | 0.647 |
|  |  | Difficulty maintaining sleep | 47 (4.8%) | 36 (4.5%) | 11 (6.1%) | 0.358 | 43 (5.0%) | 4 (3.7%) | 0.567 |
|  |  | Requires specific interventions | 32 (3.3%) | 20 (2.5%) | 12 (6.7%) | 0.004 | 31 (3.6%) | 1 (0.9%) | 0.245 |
|  |  | Early awakening | 23 (2.4%) | 20 (2.5%) | 3 (1.7%) | 0.784 | 20 (2.3%) | 3 (2.8%) | 0.734 |
|  | Pica | | 30 (3.1%) | 25 (3.1%) | 5 (2.8%) | 0.810 | 27 (3.1%) | 3 (2.8%) | 1.000 |
|  | Tic disorders | | 14 (1.4%) | 13 (1.6%) | 1 (0.6%) | 0.486 | 7 (0.8%) | 7 (6.5%) | <0.001 |
| Physical diseases | Overweight or obesity | | 124 (12.7%) | 110 (13.8%) | 14 (7.8%) | - | 106 (12.2%) | 18 (16.7%) | - |
|  |  | Overweight | 71 (7.3%) | 65 (8.2%) | 6 (3.4%) | 0.025 | 58 (6.7%) | 13 (12.0%) | 0.043 |
|  |  | Obesity | 53 (5.4%) | 45 (5.6%) | 8 (4.5%) | 0.530 | 48 (5.5%) | 5 (4.6%) | 0.697 |
|  | Gastrointestinal issues | | 126 (12.9%) | 88 (11.0%) | 38 (21.2%) | <0.001 | 116 (13.4%) | 10 (9.3%) | 0.230 |
|  |  | Functional constipation | 103 (10.6%) | 67 (8.4%) | 36 (20.1%) | <0.001 | 95 (10.9%) | 8 (7.4%) | 0.259 |
|  |  | Functional bloating | 18 (1.8%) | 15 (1.9%) | 3 (1.7%) | 1.000 | 18 (2.1%) | 0 (0.0%) | 0.247 |
|  |  | Functional diarrhea | 6 (0.6%) | 6 (0.8%) | 0 (0.0%) | 0.599 | 5 (0.6%) | 1 (0.9%) | 0.506 |
|  |  | Functional abdominal pain | 5 (0.5%) | 5 (0.6%) | 0 (0.0%) | 0.591 | 4 (0.5%) | 1 (0.9%) | 0.444 |
|  | Allergic diseases | | 112 (11.5%) | 89 (11.2%) | 23 (12.8%) | 0.523 | 97 (11.2%) | 15 (13.9%) | 0.404 |
|  |  | Allergic dermatitis or eczema | 70 (7.2%) | 55 (6.9%) | 15 (8.4%) | 0.488 | 64 (7.4%) | 6 (5.6%) | 0.490 |
|  |  | Allergic rhinitis | 40 (4.1%) | 32 (4.0%) | 8 (4.5%) | 0.782 | 32 (3.7%) | 8 (7.4%) | 0.073 |
|  |  | Bronchial asthma | 9 (0.9%) | 7 (0.9%) | 2 (1.1%) | 0.673 | 5 (0.6%) | 4 (3.7%) | 0.012 |
|  |  | IgA vasculitis | 2 (0.2%) | 2 (0.3%) | 0 (0.0%) | - | 2 (0.2%) | 0 (0.0%) | - |
|  | Febrile seizures | | 35 (3.6%) | 29 (3.6%) | 6 (3.4%) | 0.852 | 32 (3.7%) | 3 (2.8%) | 0.789 |
|  | Epilepsy | | 7 (0.7%) | 4 (0.5%) | 3 (1.7%) | 0.120 | 6 (0.7%) | 1 (0.9%) | 0.561 |
| Other problems | Food selectivity | | 439 (45.0%) | 368 (46.2%) | 71 (39.7%) | 0.114 | 391 (45.0%) | 48 (44.4%) | 0.906 |
|  |  | Resistance to green vegetables | 346 (35.5%) | 292 (36.6%) | 54 (30.2%) | 0.102 | 312 (35.9%) | 34 (31.5%) | 0.361 |
|  |  | Resistance to root vegetables | 91 (9.3%) | 80 (10.0%) | 11 (6.1%) | 0.106 | 85 (9.8%) | 6 (5.6%) | 0.153 |
|  |  | Resistance to eggs | 81 (8.3%) | 74 (9.3%) | 7 (3.9%) | 0.019 | 71 (8.2%) | 10 (9.3%) | 0.701 |
|  |  | Resistance to meat | 76 (7.8%) | 63 (7.9%) | 13 (7.3%) | 0.772 | 68 (7.8%) | 8 (7.4%) | 0.876 |
|  |  | Resistance to fruits | 51 (5.2%) | 38 (4.8%) | 13 (7.3%) | 0.175 | 44 (5.1%) | 7 (6.5%) | 0.534 |
|  |  | Resistance to dairy products | 26 (2.7%) | 24 (3.0%) | 2 (1.1%) | 0.202 | 23 (2.6%) | 3 (2.8%) | 1.000 |
|  | Behavioral problems | | 148 (15.2%) | 123 (15.4%) | 25 (14.0%) | 0.621 | 130 (15.0%) | 18 (16.7%) | 0.644 |
|  |  | Aggressive behavior | 111 (11.4%) | 93 (11.7%) | 18 (10.1%) | 0.539 | 97 (11.2%) | 14 (13.0%) | 0.581 |
|  |  | Self-injurious behavior | 53 (5.4%) | 40 (5.0%) | 13 (7.3%) | 0.231 | 45 (5.2%) | 8 (7.4%) | 0.336 |
|  | Developmental regression | | 139 (14.2%) | 112 (14.1%) | 27 (15.1%) | 0.721 | 120 (13.8%) | 19 (17.6%) | 0.291 |
|  | Swallowing or chewing problems | | 30 (3.1%) | 25 (3.1%) | 5 (2.8%) | 0.810 | 27 (3.1%) | 3 (2.8%) | 1.000 |
|  | Offensive language | | 15 (1.5%) | 13 (1.6%) | 2 (1.1%) | 1.000 | 7 (0.8%) | 8 (7.4%) | <0.001 |

For comparisons involving categorical variables with expected cell counts < 5, Fisher‘s exact test was used instead of the chi-square test, and the corresponding *p*-values are reported. For conditions with extremely low overall frequency (e.g., IgA vasculitis, total n = 2), *p*-values are not displayed due to insufficient sample size for meaningful comparison.
