# Supplementary material for: Associations Between Comorbidities, Developmental Status, and Disease Severity in Children With Autism Spectrum Disorder: A Multicenter Cross‐Sectional Study in China
Source: Autism Res. 2026 Apr 13;19(6):e70253. doi: 10.1002/aur.70253 (PMC13276685; doi:10.1002/aur.70253)
Supplement: Supplementary file 5 — Table S5: Supporting Information. [file AUR-19-0-s002.docx]

| Comorbidities  (n=762) | CARS | | | | | | | | | |
| --- | --- | --- | --- | --- | --- | --- | --- | --- | --- | --- |
|  | Model 1 | | | | Model 2 | | | Model 3 | | |
|  | Beta | | 95%CI | *p* | Beta | 95%CI | *p* | Beta | 95%CI | *p* |
| IDD | 3.100 | | 2.284, 3.916 | <0.001 | 3.051 | 2.231, 3.870 | <0.001 | 2.953 | 2.132, 3.775 | <0.001 |
| Food selectivity | 0.883 | | 0.342, 1.424 | 0.001 | 0.875 | 0.334, 1.417 | 0.002 | 0.907 | 0.367, 1.448 | 0.001 |
| Insomnia disorder | 0.636 | | -0.070, 1.342 | 0.078 | 0.611 | -0.095, 1.317 | 0.090 | 0.610 | -0.095, 1.314 | 0.091 |
| Developmental regression | 1.103 | | 0.351, 1.855 | 0.004 | 1.100 | 0.349, 1.851 | 0.004 | 1.087 | 0.338, 1.837 | 0.005 |
| Behavioral problems | 0.746 | | 0.002, 1.490 | 0.050 | 0.774 | 0.031, 1.518 | 0.041 | 0.748 | 0.006, 1.490 | 0.048 |
| Overweight or obesity | 0.791 | | -0.018, 1.600 | 0.056 | 0.816 | 0.005, 1.626 | 0.049 | 0.781 | -0.028, 1.590 | 0.059 |
| Gastrointestinal issues | 0.000 | | -0.823, 0.822 | >0.999 | -0.048 | -0.873, 0.777 | 0.909 | -0.084 | -0.910, 0.741 | 0.841 |
| Allergic diseases | -0.238 | | -1.088, 0.612 | 0.583 | -0.255 | -1.104, 0.593 | 0.556 | -0.156 | -1.006, 0.694 | 0.720 |
| Febrile seizures | 0.945 | | -0.473, 2.363 | 0.192 | 1.047 | -0.372, 2.466 | 0.149 | 1.033 | -0.382, 2.449 | 0.153 |
| Pica | 1.466 | | -0.085, 3.018 | 0.064 | 1.472 | -0.078, 3.022 | 0.063 | 1.270 | -0.284, 2.824 | 0.110 |
| Swallowing or chewing problems | 1.445 | | -0.174, 3.064 | 0.081 | 1.424 | -0.193, 3.041 | 0.085 | 1.493 | -0.118, 3.104 | 0.070 |
| Offensive language | -2.958 | | -5.225, -0.690 | 0.011 | -2.651 | -4.950, -0.352 | 0.024 | -2.732 | -5.026, -0.437 | 0.020 |
| Tic disorders | 0.916 | | -1.360, 3.192 | 0.430 | 1.197 | -1.092, 3.486 | 0.306 | 1.082 | -1.211, 3.375 | 0.355 |
| Epilepsy | -0.043 | | -3.407, 3.321 | 0.980 | 0.013 | -3.362, 3.387 | 0.994 | -0.128 | -3.498, 3.242 | 0.941 |
| GDS scales  (n=607) | Model 1 | | | | Model 2 | | | Model 3 | | |
|  | Beta | 95%CI | | *p* | Beta | 95%CI | *p* | Beta | 95%CI | *p* |
| Adaptive behavior | -0.093 | -0.111, -0.075 | | <0.001 | -0.092 | -0.111, -0.073 | <0.001 | -0.088 | -0.107, -0.068 | <0.001 |
| Gross motor | -0.072 | -0.093, -0.052 | | <0.001 | -0.069 | -0.091, -0.047 | <0.001 | -0.067 | -0.089, -0.045 | <0.001 |
| Fine motor | -0.062 | -0.079, -0.046 | | <0.001 | -0.060 | -0.077, -0.043 | <0.001 | -0.057 | -0.074, -0.039 | <0.001 |
| Language | -0.101 | -0.118, -0.083 | | <0.001 | -0.100 | -0.118, -0.083 | <0.001 | -0.095 | -0.113, -0.077 | <0.001 |
| Personal-social behavior | -0.117 | -0.139, -0.096 | | <0.001 | -0.114 | -0.136, -0.092 | <0.001 | -0.109 | -0.131, -0.087 | <0.001 |
| Wechsler scales  (n=116) | Model 1 | | | | Model 2 | | | Model 3 | | |
|  | Beta | 95%CI | | *p* | Beta | 95%CI | *p* | Beta | 95%CI | *p* |
| Normal Range | - | - | | *-* | - | - | *-* | - | - | *-* |
| Borderline | -0.077 | -2.734, 2.580 | | 0.955 | -0.112 | -2.764, 2.541 | 0.934 | -0.214 | -2.944, 2.517 | 0.878 |
| Intellectual disability | 4.033 | 2.065, 6.001 | | <0.001 | 3.763 | 1.797, 5.728 | <0.001 | 3.170 | 1.138, 5.202 | 0.003 |

**Table S5 All generalized linear models for the relationship between comorbidities, developmental status and CARS scores in ASD children (complete case analysis)**

For the comorbidities–CARS models, Model 1 was unadjusted. Model 2 was adjusted for sex and age. Model 3 was additionally adjusted for premature birth, paternal age at conception, family history of mental illness, and gestational hypertension.

For the GDS–CARS and Wechsler–CARS models, Model 1 was unadjusted. Model 2 was adjusted for sex, age, premature birth, paternal age at conception, family history of mental illness, and gestational hypertension. Model 3 was further adjusted for all covariates in Model 2, plus food selectivity, developmental regression, offensive language, and behavioral problems.

The covariates food selectivity, developmental regression, offensive language, and behavioral problems were identified as comorbidities associated with CARS scores in the comorbidities–CARS models.

Abbreviations: CARS, Childhood Autism Rating Scale; GDS, Gesell Developmental Schedule.
