# Supplementary material for: Associations Between Comorbidities, Developmental Status, and Disease Severity in Children With Autism Spectrum Disorder: A Multicenter Cross‐Sectional Study in China
Source: Autism Res. 2026 Apr 13;19(6):e70253. doi: 10.1002/aur.70253 (PMC13276685; doi:10.1002/aur.70253)
Supplement: Supplementary file 6 — Table S6: Supporting Information. [file AUR-19-0-s001.docx]

| Comorbidities | Comorbidities–CARS model 3 | | | | | |
| --- | --- | --- | --- | --- | --- | --- |
|  | Total (n=1279) | | | < 6 years (n=1141) | | |
|  | Beta | 95%CI | *p* | Beta | 95%CI | *p* |
| IDD | 2.317 | 1.662, 2.973 | <0.001 | 1.881 | 1.178, 2.583 | <0.001 |
| Food selectivity | 0.879 | 0.437, 1.322 | <0.001 | 0.711 | 0.257, 1.166 | 0.002 |
| Insomnia disorder | 1.026 | 0.439, 1.614 | <0.001 | 1.012 | 0.405, 1.618 | 0.001 |
| Developmental regression | 1.313 | 0.709, 1.918 | <0.001 | 1.243 | 0.616, 1.870 | <0.001 |
| Behavior problems | 0.531 | -0.094, 1.156 | 0.096 | 0.354 | -0.296, 1.005 | 0.286 |
| Overweight or obesity | 0.432 | -0.230, 1.094 | 0.201 | 0.312 | -0.371, 0.996 | 0.371 |
| Gastrointestinal issues | 0.151 | -0.523, 0.824 | 0.661 | 0.253 | -0.432, 0.937 | 0.470 |
| Allergic diseases | 0.040 | -0.665, 0.746 | 0.910 | 0.303 | -0.425, 1.031 | 0.415 |
| Febrile seizures | 0.811 | -0.261, 1.882 | 0.138 | 0.661 | -0.448, 1.771 | 0.243 |
| Pica | 2.016 | 0.832, 3.200 | <0.001 | 1.885 | 0.711, 3.060 | 0.002 |
| Swallowing or chewing problems | 0.937 | -0.396, 2.270 | 0.169 | 0.574 | -0.776, 1.923 | 0.405 |
| Offensive language | -2.667 | -4.611, -0.722 | 0.007 | -2.892 | -5.323, -0.461 | 0.020 |
| Tic disorders | -0.563 | -2.629, 1.503 | 0.593 | -1.618 | -4.199, 0.963 | 0.219 |
| Epilepsy | 0.935 | -1.271, 3.141 | 0.406 | 0.494 | -2.074, 3.061 | 0.706 |
| GDS scales | GDS–CARS model 3 | | | | | |
|  | Total (n=1279) | | | < 6 years (n=1141) | | |
|  | Beta | 95%CI | *p* | Beta | 95%CI | *p* |
| Adaptive behavior | -0.070 | -0.086, -0.055 | <0.001 | -0.069 | -0.084, -0.054 | <0.001 |
| Gross motor | -0.043 | -0.060, -0.027 | <0.001 | -0.045 | -0.061, -0.029 | <0.001 |
| Fine motor | -0.043 | -0.057, -0.030 | <0.001 | -0.042 | -0.055, -0.028 | <0.001 |
| Language | -0.067 | -0.081, -0.053 | <0.001 | -0.065 | -0.080, -0.051 | <0.001 |
| Personal-social behavior | -0.059 | -0.076, -0.042 | <0.001 | -0.058 | -0.075, -0.041 | <0.001 |

**Table S6** **Associations of comorbidities, GDS, and CARS: generalized linear model 3 for all ASD children and the subgroup aged < 6 years**

For the comorbidities–CARS model, Model 3 was adjusted for sex, age, premature birth, paternal age at conception, family history of mental illness, and gestational hypertension.

For the GDS–CARS model, Model 3 was adjusted for sex, age, premature birth, paternal age at conception, family history of mental illness, gestational hypertension, food selectivity, developmental regression, offensive language, pica, and insomnia disorder.

Abbreviations: CARS, Childhood Autism Rating Scale; IDD, Intellectual developmental disorders; GDS, Gesell Developmental Schedule.
