# Supplementary material for: Associations Between Comorbidities, Developmental Status, and Disease Severity in Children With Autism Spectrum Disorder: A Multicenter Cross‐Sectional Study in China
Source: Autism Res. 2026 Apr 13;19(6):e70253. doi: 10.1002/aur.70253 (PMC13276685; doi:10.1002/aur.70253)
Supplement: Supplementary file 7 — Table S7: Supporting Information. [file AUR-19-0-s004.docx]

| Comorbidities | Comorbidities–CARS model 3 | | | | | |
| --- | --- | --- | --- | --- | --- | --- |
|  | Total (n=1279) | | | ≥ 6 years (n=138) | | |
|  | Beta | 95%CI | *p* | Beta | 95%CI | *p* |
| IDD | 2.317 | 1.662, 2.973 | <0.001 | 4.320 | 2.326, 6.314 | <0.001 |
| Food selectivity | 0.879 | 0.437, 1.322 | <0.001 | 2.165 | 0.482, 3.849 | 0.013 |
| Insomnia disorder | 1.026 | 0.439, 1.614 | <0.001 | 1.314 | -0.849, 3.478 | 0.236 |
| Developmental regression | 1.313 | 0.709, 1.918 | <0.001 | 1.656 | -0.499, 3.811 | 0.134 |
| Behavior problems | 0.531 | -0.094, 1.156 | 0.096 | 2.021 | -0.128, 4.170 | 0.068 |
| Overweight or obesity | 0.432 | -0.230, 1.094 | 0.201 | 1.050 | -1.338, 3.438 | 0.390 |
| Gastrointestinal issues | 0.151 | -0.523, 0.824 | 0.661 | -0.819 | -3.581, 1.943 | 0.562 |
| Allergic diseases | 0.040 | -0.665, 0.746 | 0.910 | -1.941 | -4.496, 0.614 | 0.139 |
| Febrile seizures | 0.811 | -0.261, 1.882 | 0.138 | 1.117 | -2.723, 4.957 | 0.570 |
| Pica | 2.016 | 0.832, 3.200 | <0.001 | 4.428 | -2.530, 11.39 | 0.215 |
| Swallowing or chewing problems | 0.937 | -0.396, 2.270 | 0.169 | 4.142 | -1.566, 9.850 | 0.157 |
| Offensive language | -2.667 | -4.611, -0.722 | 0.007 | -1.922 | -5.841, 1.996 | 0.338 |
| Tic disorders | -0.563 | -2.629, 1.503 | 0.593 | 0.420 | -3.714, 4.554 | 0.843 |
| Epilepsy | 0.935 | -1.271, 3.141 | 0.406 | 2.205 | -2.828, 7.238 | 0.392 |
| Wechsler scales | Wechsler–CARS model 3 | | | | | |
|  | Total (n=1279) | | | ≥ 6 years (n=138) | | |
|  | Beta | 95%CI | *p* | Beta | 95%CI | *p* |
| Normal Range | - | - | *-* | - | - | *-* |
| Borderline | 0.749 | -1.349, 2.846 | 0.485 | -0.451 | -3.851, 2.950 | 0.795 |
| Intellectual disability | 3.011 | 1.417, 4.605 | <0.001 | 3.809 | 1.189, 6.429 | 0.005 |

**Table S7** **Associations of comorbidities, Wechsler, and CARS: generalized linear model 3 for all ASD children and the subgroup aged ≥ 6 years**

For the comorbidities–CARS model, Model 3 for both groups was adjusted for sex, age, premature birth, paternal age at conception, family history of mental illness, and gestational hypertension.

For the Wechsler–CARS model, Model 3 for the total group was adjusted for sex, age, premature birth, paternal age at conception, family history of mental illness, gestational hypertension, food selectivity, developmental regression, offensive language, pica, and insomnia disorder.

For the Wechsler–CARS model, Model 3 for the ≥ 6 years group was adjusted for sex, age, premature birth, paternal age at conception, family history of mental illness, gestational hypertension, and food selectivity.

In the ≥ 6 years group, only food selectivity was included in the Wechsler–CARS Model 3, as it was the sole comorbidity found to be associated with CARS scores in the comorbidities–CARS models for this subgroup.

Abbreviations: CARS, Childhood Autism Rating Scale; IDD, Intellectual developmental disorders.
